# Effects of Defects on the Mechanical Properties of Kinked Silicon Nanowires

**DOI:** 10.1186/s11671-017-1970-7

**Published:** 2017-03-09

**Authors:** Yun Chen, Cheng Zhang, Liyi Li, Chia-Chi Tuan, Xin Chen, Jian Gao, Yunbo He, Ching-Ping Wong

**Affiliations:** 1 School of Electromechanical Engineering, Guangdong University of Technology, Guangzhou, 510006 China; 2 Key Laboratory of Mechanical Equipment Manufacturing and Control Technology of Ministry of Education, Guangdong University of Technology, Guangzhou, 510006 China; 3 School of Materials Science and Engineering, Georgia Institute of Technology, 711 Ferst Drive, Atlanta, GA 30332 USA; 4 School of Materials Science and Engineering, Southeast University, Nanjing, 211189 China; 5 School of Engineering, The Chinese University of Hong Kong, Shatin, Hong Kong

**Keywords:** Kinked silicon nanowires, Effects of defects, Mechanical properties, Molecular dynamics simulation

## Abstract

Kinked silicon nanowires (KSiNWs) have many special properties that make them attractive for a number of applications. The mechanical properties of KSiNWs play important roles in the performance of sensors. In this work, the effects of defects on the mechanical properties of KSiNWs are studied using molecular dynamics simulations and indirectly validated by experiments. It is found that kinks are weak points in the nanowire (NW) because of inharmonious deformation, resulting in a smaller elastic modulus than that of straight NWs. In addition, surface defects have more significant effects on the mechanical properties of KSiNWs than internal defects. The effects of the width or the diameter of the defects are larger than those of the length of the defects. Overall, the elastic modulus of KSiNWs is not sensitive to defects; therefore, KSiNWs have a great potential as strain or stress sensors in special applications.

**Electronic supplementary material:**

The online version of this article (doi:10.1186/s11671-017-1970-7) contains supplementary material, which is available to authorized users.

## Background

Kinked silicon nanowires (KSiNWs) can trap more photons [1], enhance thermal isolation [2–4], strengthen the strain effect [5, 6], and be feasible for biosensor detection [7–9], widely broadening its application areas [10–12]. However, such applications of KSiNWs are notably affected by their mechanical properties [13].

Several experimental and modeling studies have been conducted to identify the dependence of Young’s modulus and yield strength of nanowires (NWs) on factors including temperature, loading rate or strain rate, geometry, and size [14–18]. As KSiNWs are recently developed, only a few studies have reported their mechanical properties. Jiang et al. studied the size dependence of the Young’s modulus in KSiNWs using both molecular dynamics (MD) simulations and finite element methods and found that the Young’s modulus is sensitive to its arm length; specifically, it decreases rapidly with increasing arm length [19]. In addition, they also found that KSiNWs can behave like springs [5]. Jing et al. also studied the effects of geometry on the mechanical properties of KSiNWs and found that the fracture stress decreases as the periodic length increases [6]. These useful findings can provide insights into KSiNW fabrication and development of their applications.

However, KSiNWs—either grown by the classic metal catalytic vapor–liquid–solid method [10, 20–22] or fabricated by the newly developed metal-assisted chemical etching method [23]—usually have defects that may affect their functionality. As it is hard to control the location and size of the defect in a KSiNW—difficulties arise in terms of sample clamping, precise manipulation and alignment, and accurate measurement of the response (e.g., force, displacement) [15, 24]—little research about effects of defects on the mechanical properties of KSiNWs has been reported. Herein, we report the effects of various defects on the mechanical properties of KSiNWs by a molecular dynamics modeling study.

## Methods

**Fig. 1:**
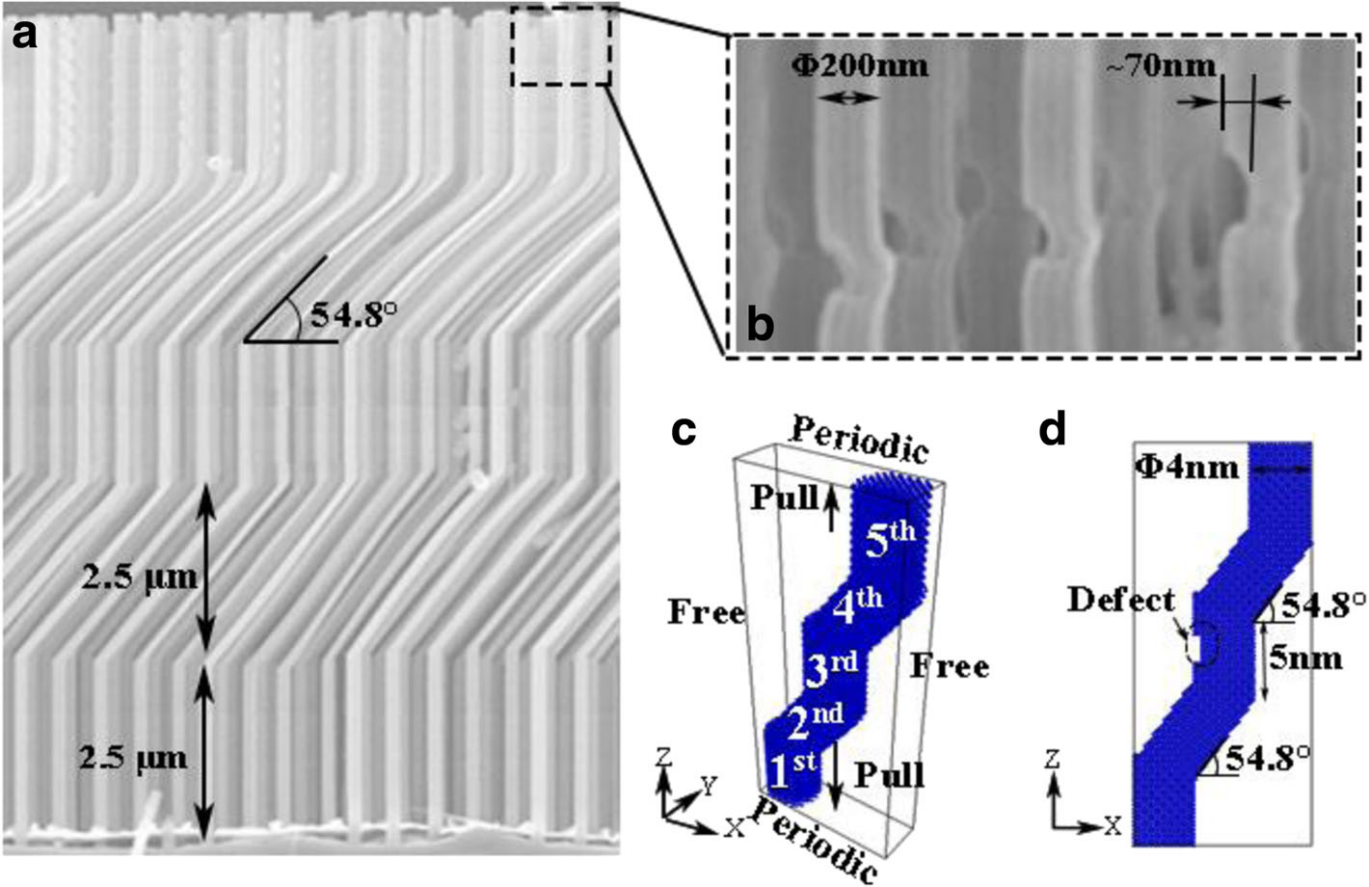
The geometry of KSiNWs. **a** KSiNWs fabricated by alternating MACE. **b** Typical defects in KSiNWs. **c** Molecular dynamic model of perfect KSiNWs. The periodic boundary condition was applied in the *axial direction*, and the free boundary condition was applied in the *lateral direction*. **d** Surface defects in the model of KSiNWs. *Both* ends of the KSiNWs were pulled simultaneously

KSiNWs (Fig. 1a) were fabricated by the alternating metal-assisted chemical etching (Alternating MACE) method in our previous report [23]. First, patterns of polystyrene (PS) microspheres were formed on Si (P type, single-crystalline, (100)-oriented, boron doped, resistivity 1–10 Ω cm) through a self-assembly process [25]. Then, 3-nm-thick titanium (Ti) and 30-nm-thick gold (Au) were deposited as the catalysts on Si. After that, Si was immersed into etchant *A* that consisted of 20 ml deionized (DI) water, 2 ml hydrogen peroxide (H_2_O_2_), and 10 ml hydrofluoric acid (HF) for 5 min, when straight NWs of length 2.5 μm were formed. Then, the Si was removed from etchant *A* and immersed into another etchant solution *B* that consisted of 15 ml DI water, 5 ml glycerol, 2 ml H_2_O_2_, and 10 ml HF for 10 min to form slanted NWs. Because the etching directions were different in the two etchants, kinks were formed at the joint of different crystallographic directions or some vector combinations of them. In this case, the straight and slanted etching direction are [100] and [21-1], respectively [23]. By repeating similar procedures *A-B-A-B-A*, periodically, KSiNWs were fabricated that consisted mainly of three straight segments and two slanted segments with an angle of 54.8°.

To save computational time, the geometry size was scaled from the original. Each segment was about 5 nm in length. The cross section of each segment was 4 nm in diameter. The 3D simulation model was developed using the open-source software Lammps [26], as shown in Fig. 1c. There were 18,066 atoms for a perfect KSiNW.

From the scanning electron microscopy image (SEM, shown in Fig. 1b), cuboid-like defects can be seen in the NWs. The width of most defects is about one-third of the diameter of the KSiNWs. Therefore, in this work, various surface or internal defects were manually inserted depending on the conditions considered. The defect is denoted as location–width–length for simplification, e.g., the defect located in the middle of the third segment 0.5 nm in length and 1.0 nm in width is demonstrated as 3rd-W0.5-L1.0.

The periodic boundary condition was applied in the axial direction, and the free boundary condition was applied in the lateral direction. The atomic interactions were described using the Stillinger–Weber (SW) potential. The velocity–Verlet algorithm was employed to integrate the equations of motion. All molecular systems were equilibrated at a constant pressure of 1 atm and a temperature of 0.01 K using a constant number of particles, volume, and temperature (NVT) for 10 ps with a time step of 2 fs. The strain was then applied along the uniaxial direction to perform uniaxial tensile tests. The applied strain rate was 0.00025/ps. The strain increment was applied to the structure after every 900,000 time steps. All MD simulations were carried out at 0.1 K, and the temperature was controlled employing the Nosé–Hoover thermostat.

## Results

It is hard to directly measure the mechanical properties of KSiNWs, especially in KSiNWs with defects. The mechanical properties of straight silicon NWs have been reported by many researchers. Therefore, to verify the model, a molecular dynamics model of straight NWs was also developed using the same parameters (with different geometry parameters) and the simulation results were compared with the reported ones.

**Fig. 2:**
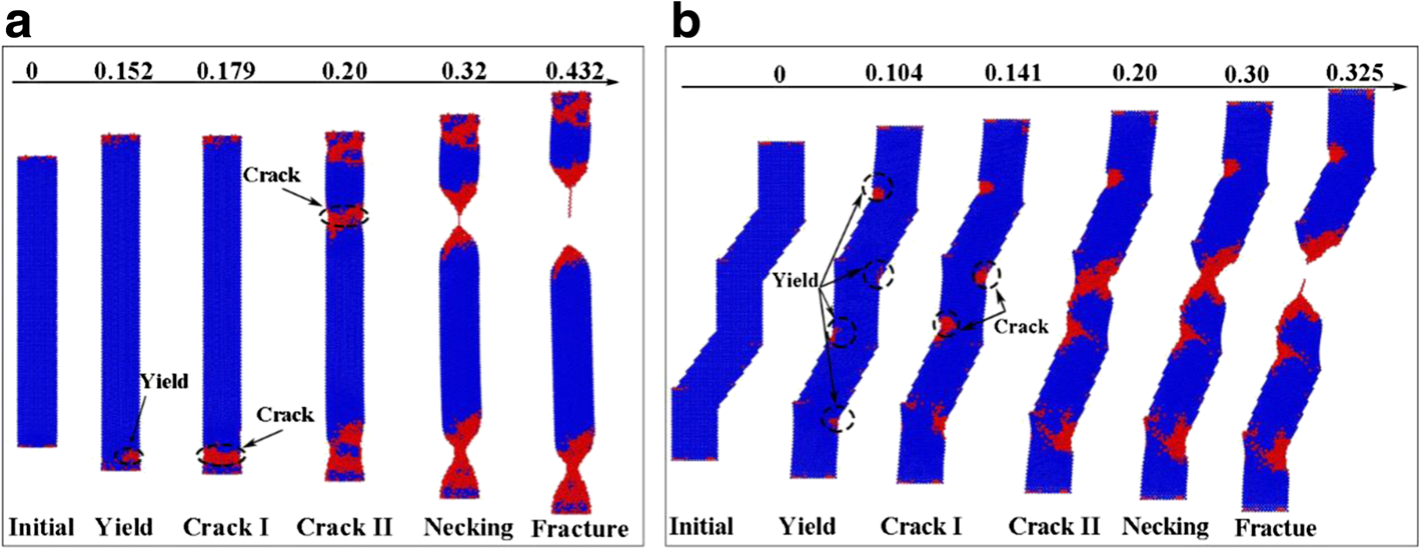
Tensioning process of NW. **a** Straight NW and **b** KSiNWs. The tensioning processes of *both straight* and KSiNWs consist of *yield*, *crack*, *necking*, and *fracture* stages

**Fig. 3:**
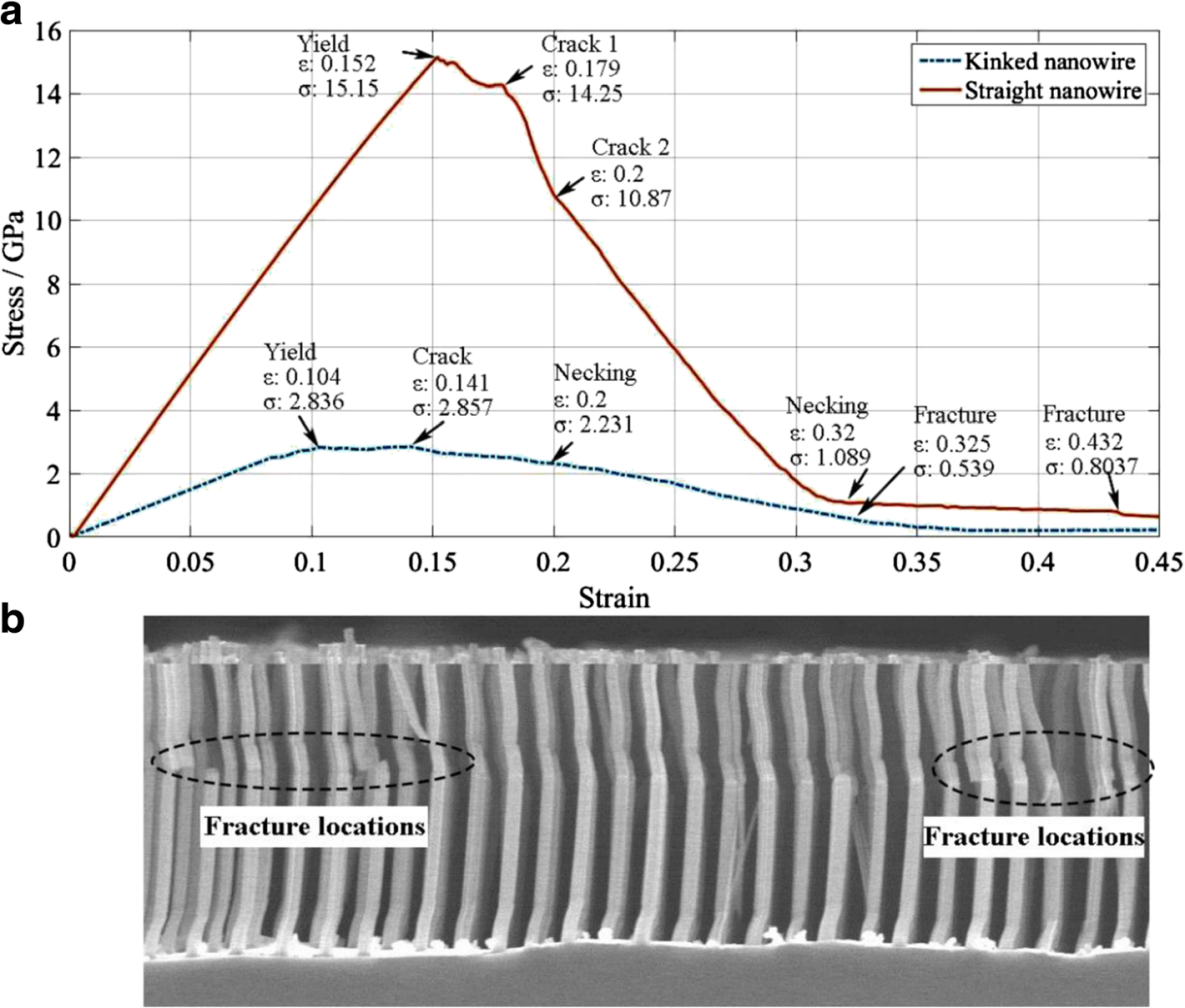
NWs during tensioning. **a** Stress–strain relationships of NWs during tensioning. **b** KSiNWs fractured at the kinks after external force excitation

The tensioning process of NWs and the relationship between stress and strain are shown in Figs. 2 and 3a, respectively. It can be seen that the yield stress and yield strain of the straight NWs are 15.15 GPa and 0.152. Thus, the elastic modulus (*ε* < 3%) can be calculated as 106.44 GPa using the commonly used equation [27]. The calculated results are consistent with the experimental results of 93–180 GPa [13–16].

$$ E=\frac{\sigma_{0.03}}{\varepsilon_{0.03}} $$

**Fig. 4:**
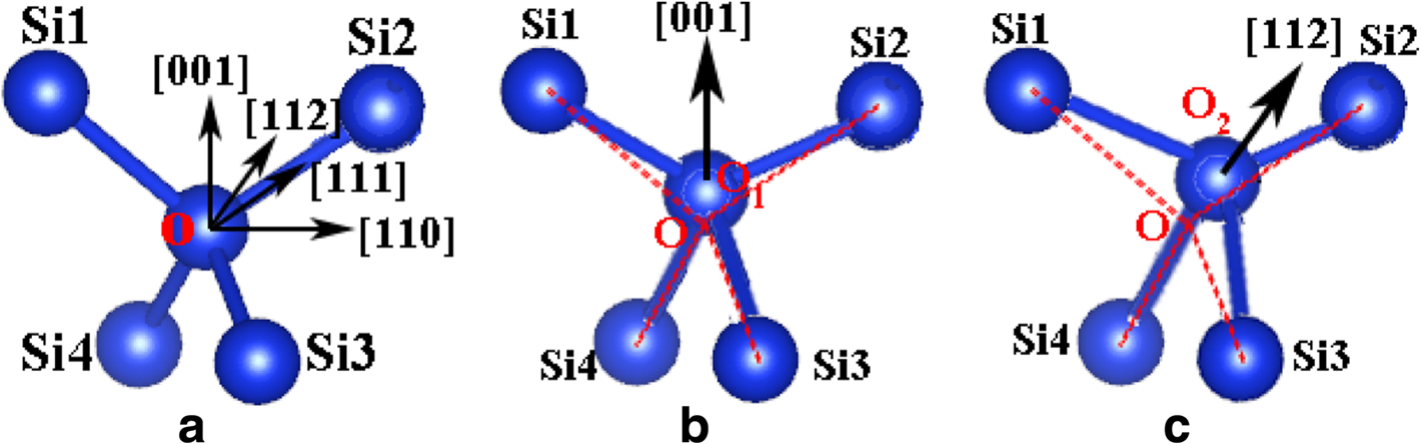
*Bond configuration* of crystalline silicon. **a** *Initial bond configuration* of crystalline silicon. *Bond deformation* of crystalline silicon when the strain is applied along **b** [001] and **c** [112]

Using the same method, the yield stress, yield strain, and elastic modulus of the KSiNWs can be identified as about 2.836 GPa, 0.104, and 29.987 GPa, respectively. It can be seen that all quantities are smaller than those of the straight NWs. This can be explained as follows:

When strain is applied along the [100] direction on the straight segments whose crystalline directions are along the [100] direction, the bond angles ∠Si1O_1_Si4, ∠Si2O_1_Si3, and ∠Si3O_1_Si4 can decrease while only ∠Si1O_1_Si2 increases [28] (Fig. 4b). However, for the slanted segments whose crystalline directions are along the [112] direction, where the strain is applied along the [112] direction, the bond angles ∠Si1O_2_Si4 and ∠Si3O_2_Si4 can decrease while both ∠Si1O_2_Si2 and ∠Si2O_2_Si3 increase (Fig. 4c), resulting in inharmonious deformation in different segments and then initially fracturing at kinks (the junction of straight and slanted segments). Consequently, the kink introduces weaker points in the NWs because of inharmonious deformation, thus, KSiNWs are weaker than the straight NWs. Experimental results that KSiNWs break at kinks after external force excitation (Fig. 3b) also confirm this. The modeling and experimental results demonstrate that the model is accurate.

The fracture strain of straight NW is about 0.179, approaching the theoretical elastic limit of silicon (17 to 20%) [29]. No hardening phenomenon is observed after it yields. The fracture stress is the same as the yield stress (14.25 GPa) which is also consistent with the experimental value (~20 GPa) [29]. However, as the KSiNWs are more spring-like, their fracture strain increases 35.58 to 0.141%, and a small hardening is observed after it yields. Its fracture stress increases slightly to 2.857 GPa.

## Discussion

### Effects of Surface Defects on the Mechanical Properties of Kinked Nanowire

#### Size of the Defect

**Fig. 5:**
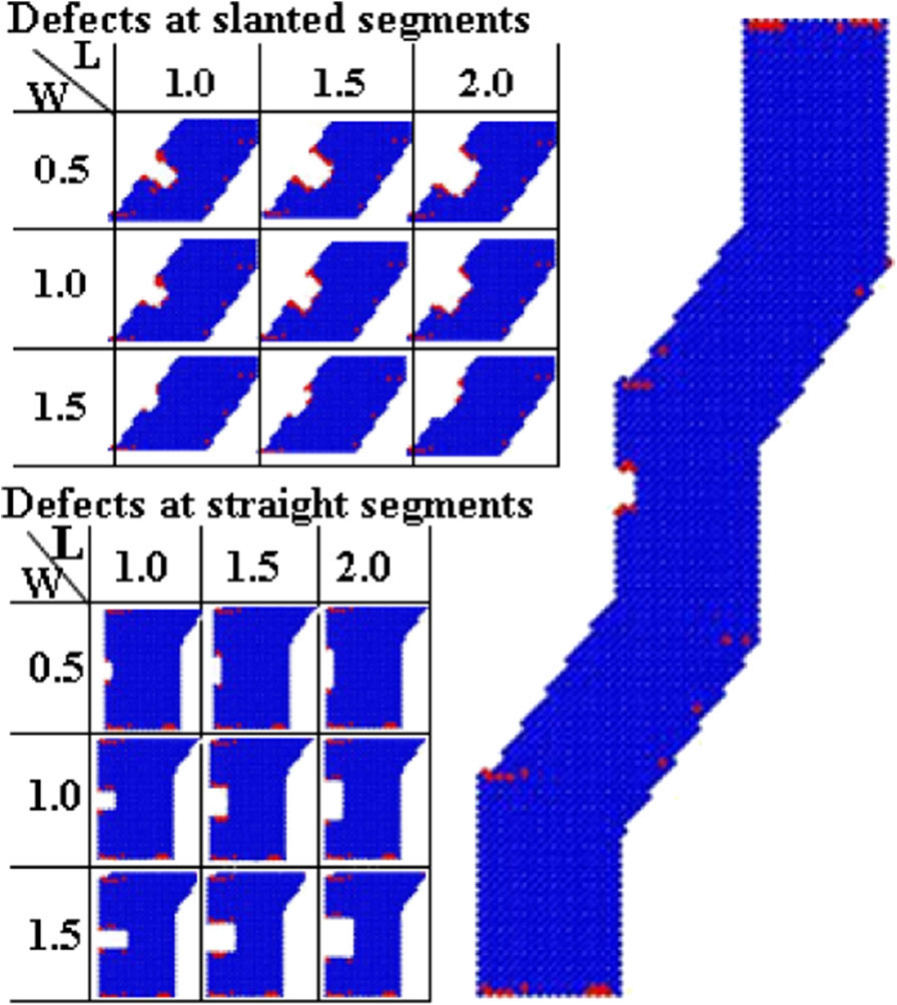
Various surface defects in KSiNWs. There are nine conditions for each segment, and 36 simulations in total for this series of studies

To study the effects of the size of the defect on the mechanical properties of the KSiNW, tensioning processes of NW with defects of different sizes were conducted, as shown in Fig. 5. The length (L) of the defect was varied as 1.0, 1.5, and 2 nm with the same width in each case, or the width (W) of the defect was varied as 0.5, 1.0, and 1.5 nm with the length kept constant. Defect locations in the 1st to 4th segment were considered. Other geometry and simulation parameters were kept the same as the control case.

**Fig. 6:**
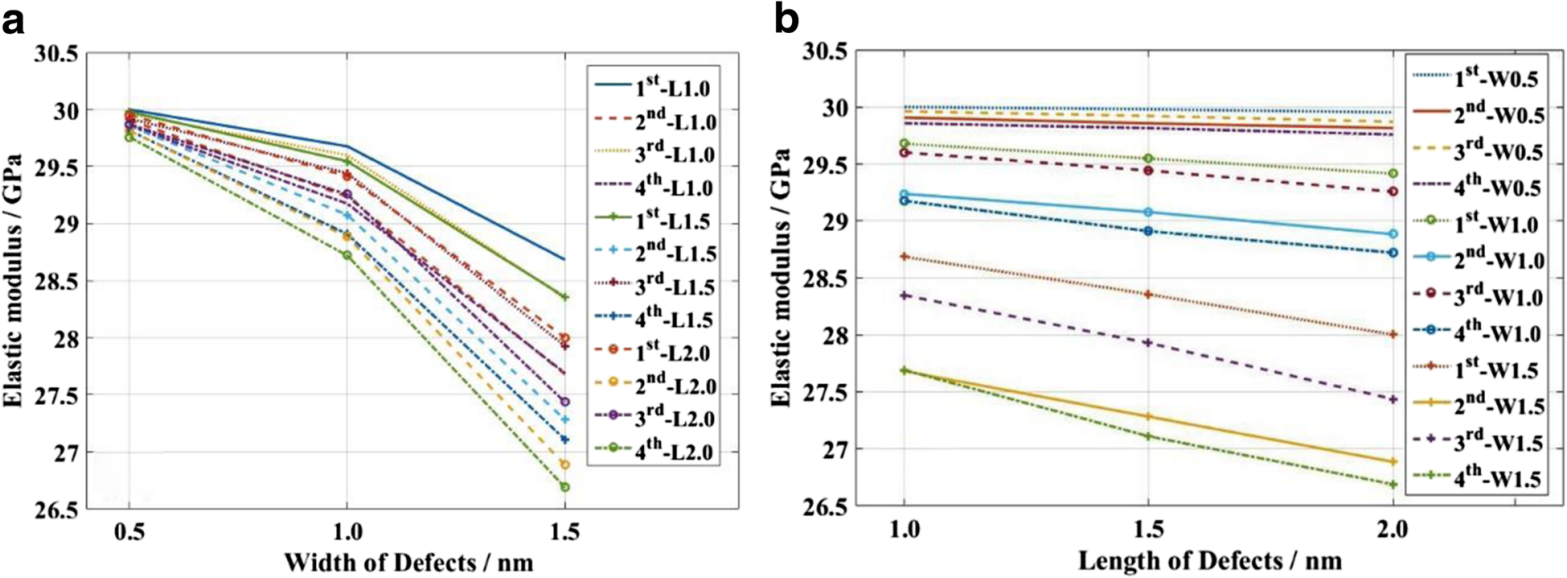
Effects of the defect size on the elastic modulus of KSiNWs. Defect locations in the 1st–4th segment were considered. **a** Effect of varying the *width* of the defect with the same *length* in each case. **b** Effect of varying the *length* of the defect with the same width in each case

The relationship between strain and stress, and the final profiles for each case are shown in the Additional file 1. The defects have notable effects on the mechanical properties of KSiNWs, especially for the relationships between stress and strain, and fracture locations. However, as for most of the applications of KSiNW, the KSiNW functions in the elastic range. Therefore, to evaluate the influence of the defects quantitatively, the elastic modulus was calculated (Fig. 6). The elastic modulus decreases when the defect size increases. The elastic modulus decreases linearly with the length of the defect. However, the elastic modulus decreases nonlinearly with the width of the defect. In addition, the elastic modulus decreases faster with the width of the defect than that with the length of the defect. This means that the width of the defect has more significant effects on the mechanical properties of KSiNWs than the length of the defect.

#### Location of the Defect

To study the effects of the location of the defect on the mechanical properties of the KSiNWs, the tensioning of NWs with defects in different segments was conducted. The locations of defects varied from the 1st to the 4th segment. Different sizes of defects with lengths ranging from 1.0 to 2 nm or widths from 0.5 to 1.5 nm were all considered, as shown in Fig. 5. Other geometry and simulation parameters were kept the same as the control case.

**Fig. 7:**
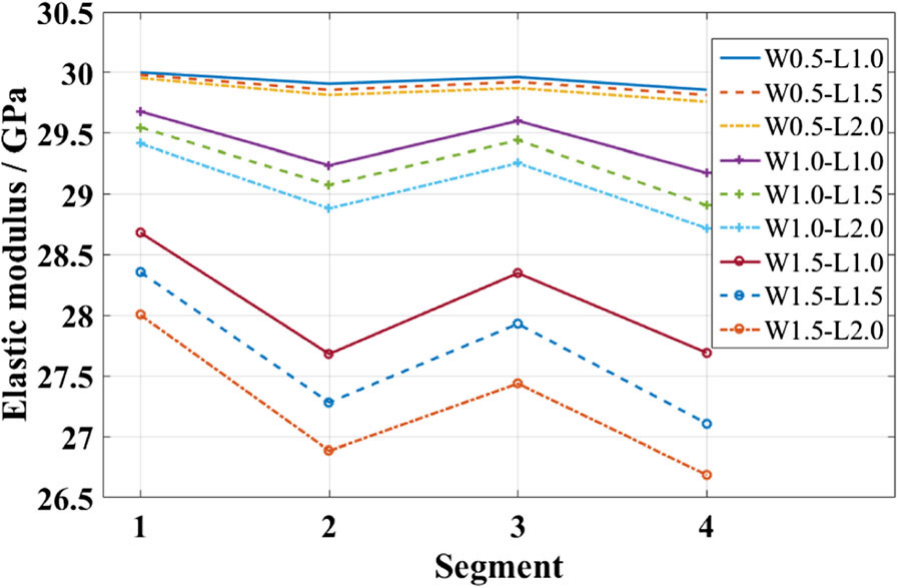
Effects of the defect location on the elastic modulus of KSiNWs. The locations of defects varied from the 1st to 4th segment. Different sizes of defects with the *length* varying from 1.0 to 2 nm or *width* varying from 0.5 to 1.5 nm were considered

Figure 7 shows the elastic moduli of KSiNWs that have defects in different segments. It can be seen that the elastic modulus decreased more when the defects were in the slanted segments (2nd and 4th segment) than when the defects were in the straight segments (1st and 3rd segment). In addition, defects in the 4th segment have the largest effects on the elastic modulus of KSiNWs in all cases.

Conclusively, when the defect width is less than 12.5% of the diameter of the KSiNW (0.5 nm in this study), irrespective of the location and length, the influence of the defect on the elastic modulus of KSiNWs is negligible. The maximum reduction of elastic modulus is less than 10% even when the size of the defect increases notably to almost half of the diameter of the segment. This means that the elastic modulus is not sensitive to the defects. It also demonstrates that KSiNWs have a great potential as strain or stress sensors in some special applications, e.g., bio-sensors [7, 30, 31].

### Effects of Internal Defects on the Mechanical Properties of Kinked Nanowire

As in most cases, the fracture locations are in the middle of the KSiNWs (3rd segment). As a result, in this section, tensioning processes of KSiNWs with different internal defects at the 3rd segment were conducted. The diameter of the internal defect was varied as 0.5, 1, and 1.5 nm while the length varied as 1, 2, and 3 nm. For simplification, they were referred to as cases 1–9: diameter × length. All other modeling and simulation parameters were kept unchanged.

**Fig. 8:**
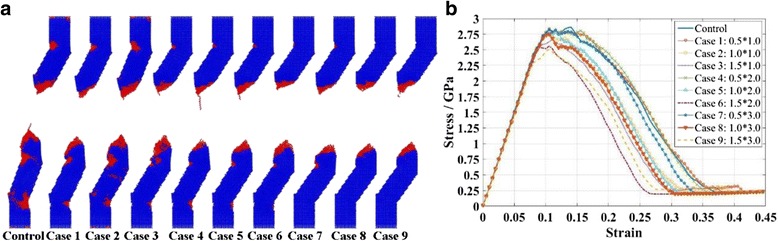
Effects of the *internal* defects on the mechanical properties of KSiNWs. **a** Final profiles of KSiNWs with *internal* defects after tensioning. All the NWs fractured at the *middle*. **b** The stress–strain relationships of KSiNWs with internal defects during tensioning. Before yielding, the stress–strain relationships are almost the same

Figure 8a shows the final tensioning profile of each NW. All the NWs fractured at the middle. It means that the internal defect has little effect on the fracture location. It can also be noted that the crack on the other kinks decreases as the size of the defect increases, while the fracture area seems to concentrate towards the middle of the NW. Figure 8b shows the stress–strain relationships of KSiNWs during tensioning. Before yield, the stress–strain relationship is almost the same. However, large internal defects cause a large reduction of fracture strength.

**Fig. 9:**
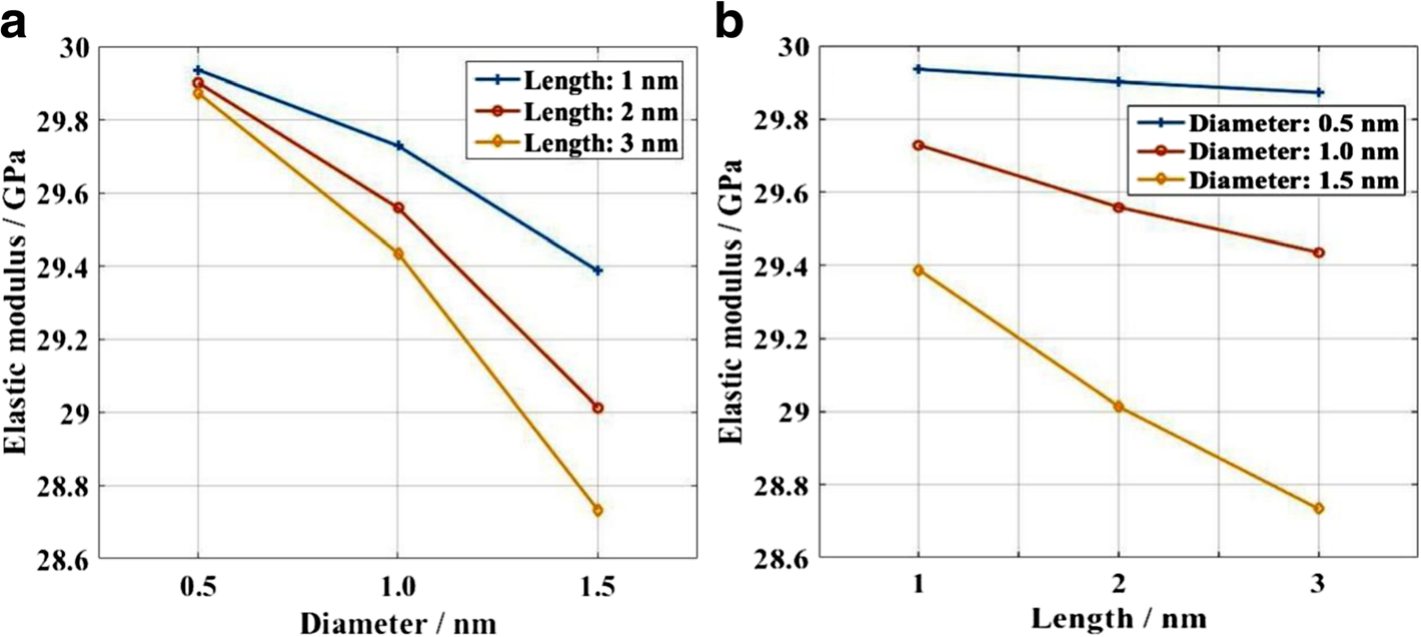
Effects of *internal* defects on the elastic modulus of KSiNWs. **a** Elastic modulus *decreases* with *increasing* diameter of *internal* defects and **b** elastic modulus *decreases* with *increasing length* of *internal* defects

Figure 9 shows the elastic modulus of KSiNWs with different internal defects. The internal defects slightly decrease the elastic modulus. The maximum reduction is only ~4%. Compared to the surface defects, the effect of internal defects is rather limited. In addition, the elastic modulus decreases linearly with both the diameter and the length of the internal defect. However, the diameter of the internal defect has a larger influence on the elastic modulus than the length of the internal defect.

## Conclusions

In this work, a molecular dynamics model was developed to study the effects of defects on the mechanical properties of KSiNWs. The simulation results were indirectly validated by experiments. It was found that KSiNWs are weaker than straight silicon NW as kinks introduce weaker points to the NWs. In addition, surface defects have more significant effects on the mechanical properties of KSiNWs than internal defects. The width of a surface defect has a more significant effect on the mechanical properties of a KSiNW than the length of a surface defect. The diameter of the internal defects has a larger influence on the elastic modulus than that the length of the internal defect. In short, the elastic modulus of KSiNWs is not sensitive to the defects. Therefore, the KSiNWs have great potential in strain or stress sensors in special applications.

## Additional File

Additional file 1: Figure S1. (a) The relationships between the strain and stress of KSiNWs with defects when tensioning. (b) Final profiles after fracture. (I) Control; (II) 1st-W0.5-L1.0; (III) 1st-W1.0-L1.0; (IV) 1st-W1.5-L1.0. **Figure S2.** (a) The relationships between the strain and stress of KSiNWs with defects when tensioning. (b) Final profiles after fracture. (I) Control; (II) 1st-W0.5-L1.5; (III) 1st-W1.0-L1.5; (IV) 1st-W1.5-L1.5. **Figure S3.** (a) The relationships between the strain and stress of KSiNWs with defects when tensioning. (b) Final profiles after fracture. (I) Control; (II) 1st-W0.5-L2.0; (III) 1st-W1.0-L2.0; (IV) 1st-W1.5-L2.0. **Figure S4.** (a) The relationships between the strain and stress of KSiNWs with defects when tensioning. (b) Final profiles after fracture. (I) Control; (II) 2nd-W0.5-L1.0; (III) 2nd-W1.0-L1.0; (IV) 2nd-W1.5-L1.0. **Figure S5.** (a) The relationships between the strain and stress of KSiNWs with defects when tensioning. (b) Final profiles after fracture. (I) Control; (II) 2nd-W0.5-L1.5; (III) 2nd-W1.0-L1.5; (IV) 2nd-W1.5-L1.5. **Figure S6.** (a) The relationships between the strain and stress of KSiNWs with defects when tensioning. (b) Final profiles after fracture. (I) Control; (II) 2nd-W0.5-L2.0; (III) 2nd-W1.0-L2.0; (IV) 2nd-W1.5-L2.0. **Figure S7.** (a) The relationships between the strain and stress of KSiNWs with defects when tensioning. (b) Final profiles after fracture. (I) Control; (II) 3rd-W0.5-L1.0; (III) 3rd-W1.0-L1.0; (IV) 3rd-W1.5-L1.0. **Figure S8.** (a) The relationships between the strain and stress of KSiNWs with defects when tensioning. (b) Final profiles after fracture. (I) Control; (II) 3rd-W0.5-L1.5; (III) 3rd-W1.0-L1.5; (IV) 3rd-W1.5-L1.5. **Figure S9.** (a) The relationships between the strain and stress of KSiNWs with defects when tensioning. (b) Final profiles after fracture. (I) Control; (II) 3rd-W0.5-L2.0; (III) 3rd-W1.0-L2.0; (IV) 3rd-W1.5-L2.0. **Figure S10.** (a) The relationships between the strain and stress of KSiNWs with defects when tensioning. (b) Final profiles after fracture. (I) Control; (II) 4th-W0.5-L1.0; (III) 4th-W1.0-L1.0; (IV) 4th-W1.5-L1.0. **Figure S11.** (a) The relationships between the strain and stress of KSiNWs with defects when tensioning. (b) Final profiles after fracture. (I) Control; (II) 4th-W0.5-L1.5; (III) 4th-W1.0-L1.5; (IV) 4th-W1.5-L1.5. **Figure S12.** (a) The relationships between the strain and stress of KSiNWs with defects when tensioning. (b) Final profiles after fracture. (I) Control; (II) 4th-W0.5-L2.0; (III) 4th-W1.0-L2.0; (IV) 4th-W1.5-L2.0. (DOCX 37176 kb)
