# Supplementary material for: Effects of Defects on the Mechanical Properties of Kinked Silicon Nanowires
Source: Nanoscale Res Lett. 2017 Mar 9;12:185. doi: 10.1186/s11671-017-1970-7 (PMC5344875; doi:10.1186/s11671-017-1970-7)
Supplement: Additional file 1: Figure S1. — (a) The relationships between the strain and stress of KSiNWs with defects when tensioning. (b) Final profiles after fracture. (I) Control; (II) 1st-W0.5-L1.0; (III) 1st-W1.0-L1.0; (IV) 1st-W1.5-L1.0. Figure S2. (a) The relationships between the strain and stress of KSiNWs with defects when tensioning. (b) Final profiles after fracture. (I) Control; (II) 1st-W0.5-L1.5; (III) 1st-W1.0-L1.5; (IV) 1st-W1.5-L1.5. Figure S3. (a) The relationships between the strain and stress of KSiNWs with defects when tensioning. (b) Final profiles after fracture. (I) Control; (II) 1st-W0.5-L2.0; (III) 1st-W1.0-L2.0; (IV) 1st-W1.5-L2.0. Figure S4. (a) The relationships between the strain and stress of KSiNWs with defects when tensioning. (b) Final profiles after fracture. (I) Control; (II) 2nd-W0.5-L1.0; (III) 2nd-W1.0-L1.0; (IV) 2nd-W1.5-L1.0. Figure S5. (a) The relationships between the strain and stress of KSiNWs with defects when tensioning. (b) Final profiles after fracture. (I) Control; (II) 2nd-W0.5-L1.5; (III) 2nd-W1.0-L1.5; (IV) 2nd-W1.5-L1.5. Figure S6. (a) The relationships between the strain and stress of KSiNWs with defects when tensioning. (b) Final profiles after fracture. (I) Control; (II) 2nd-W0.5-L2.0; (III) 2nd-W1.0-L2.0; (IV) 2nd-W1.5-L2.0. Figure S7. (a) The relationships between the strain and stress of KSiNWs with defects when tensioning. (b) Final profiles after fracture. (I) Control; (II) 3rd-W0.5-L1.0; (III) 3rd-W1.0-L1.0; (IV) 3rd-W1.5-L1.0. Figure S8. (a) The relationships between the strain and stress of KSiNWs with defects when tensioning. (b) Final profiles after fracture. (I) Control; (II) 3rd-W0.5-L1.5; (III) 3rd-W1.0-L1.5; (IV) 3rd-W1.5-L1.5. Figure S9. (a) The relationships between the strain and stress of KSiNWs with defects when tensioning. (b) Final profiles after fracture. (I) Control; (II) 3rd-W0.5-L2.0; (III) 3rd-W1.0-L2.0; (IV) 3rd-W1.5-L2.0. Figure S10. (a) The relationships between the strain and stress of KSiNWs with defects when tensioning. [file 11671_2017_1970_MOESM1_ESM.docx]

## Effects of defects on the mechanical properties of kinked silicon nanowires

Yun Chen, Cheng Zhang, Liyi Li, Chia-Chi Tuan, Xin Chen, Jian Gao, Yunbo He,

Ching-Ping Wong

1. The defects located at the 1^st^ segments. The length (L) of the defect was varied as 1.0, 1.5, and 2 nm with the same width in each case; or the width (W) of the defect was varied as 0.5, 1.0, and 1.5 nm with the length kept constant. The perfect KSiNW was used as the control. It can be seen that the defects can notably affect the fracture location and the relationship between the strain and stress, especially after yield, as shown in Figure S1-S3. In addition, when the width of defects was larger than 1.0 nm, the yield stress reduced a lot and the fracture location changed from the middle of KSiNW to the point where the defect was.


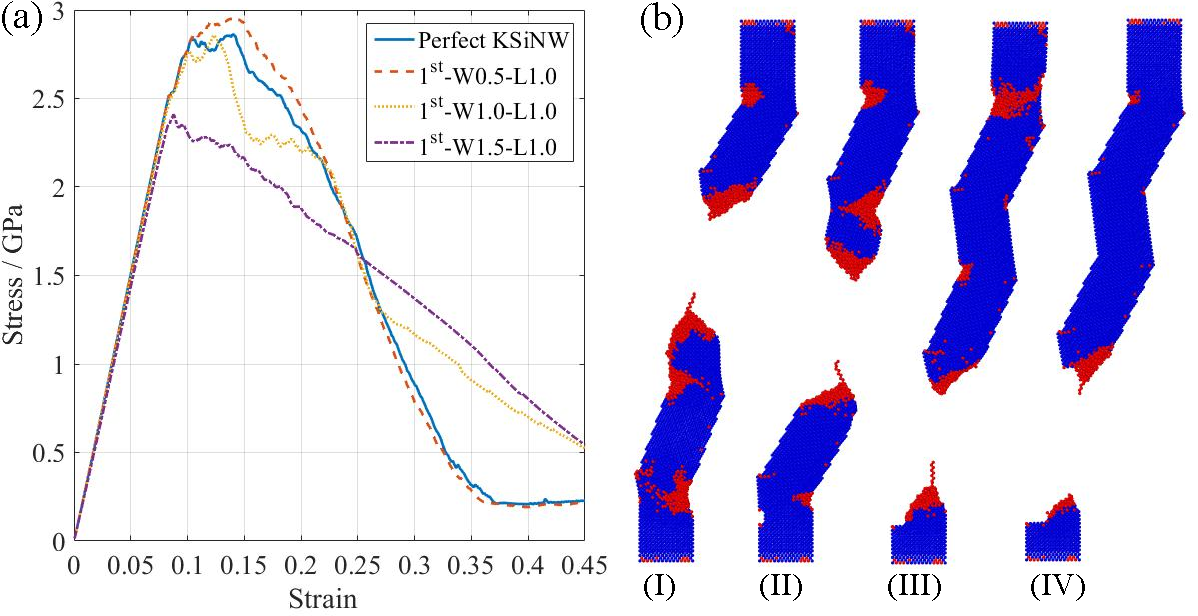


Figure S1. (a) The relationships between the strain and stress of KSiNWs with defects when tensioning. (b) Final profiles after fracture. (I) Control; (II) 1^st^-W0.5-L1.0; (III) 1^st^-W1.0-L1.0; (IV) 1^st^-W1.5-L1.0.


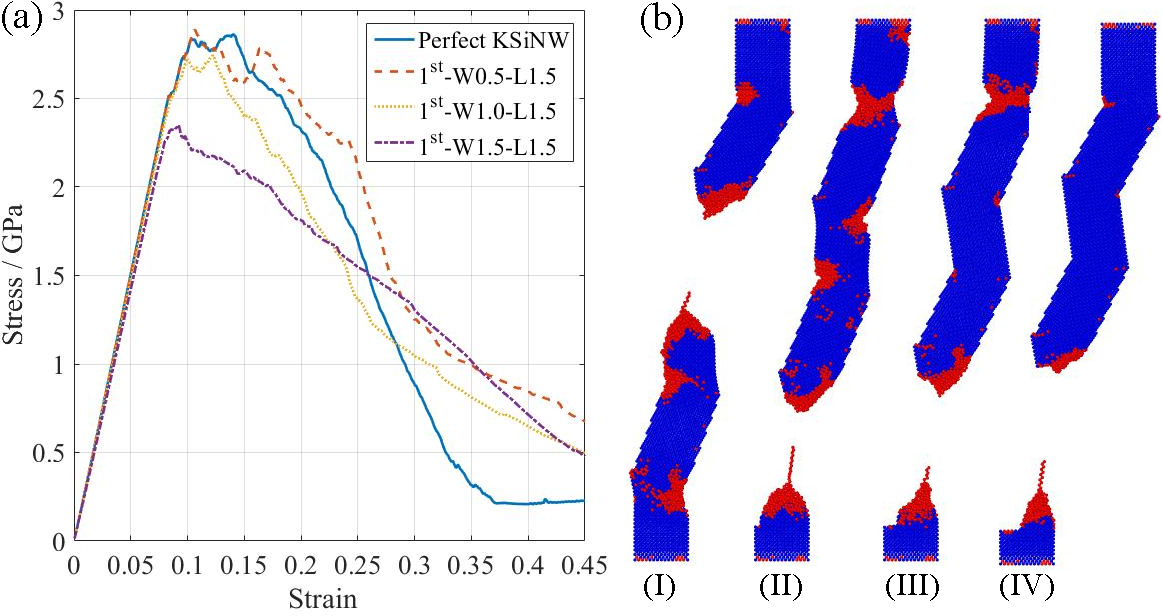


Figure S2. (a) The relationships between the strain and stress of KSiNWs with defects when tensioning. (b) Final profiles after fracture. (I) Control; (II) 1^st^-W0.5-L1.5; (III) 1^st^-W1.0-L1.5; (IV) 1^st^-W1.5-L1.5.


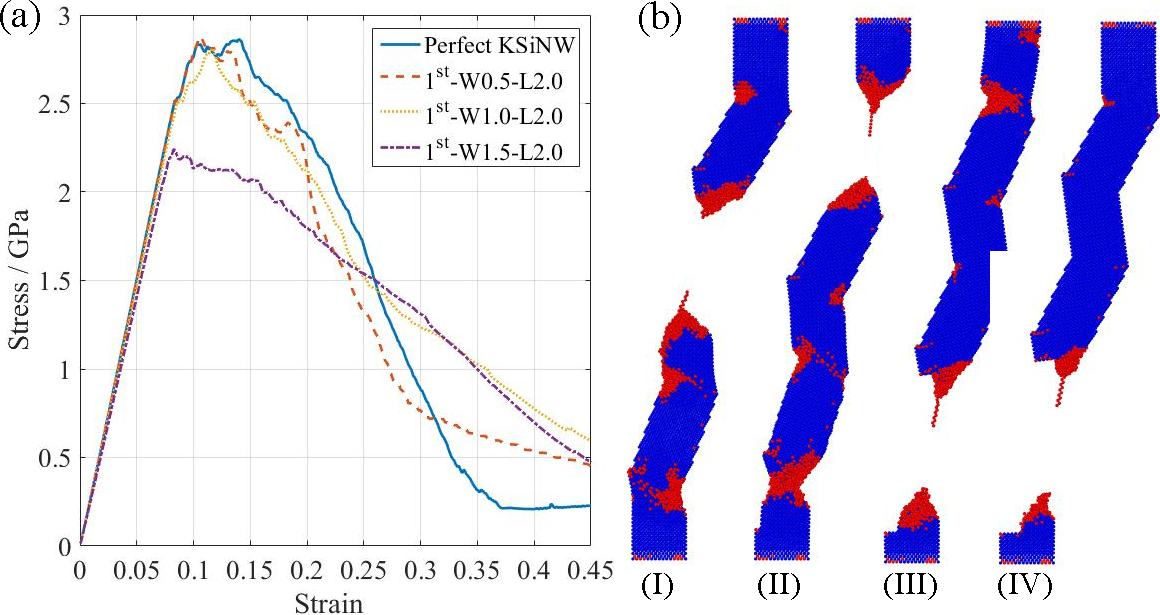


Figure S3. (a) The relationships between the strain and stress of KSiNWs with defects when tensioning. (b) Final profiles after fracture. (I) Control; (II) 1^st^-W0.5-L2.0; (III) 1^st^-W1.0-L2.0; (IV) 1^st^-W1.5-L2.0.

1. The defects located at the 2^nd^ segments. The length (L) of the defect was varied as 1.0, 1.5, and 2 nm with the same width in each case; or the width (W) of the defect was varied as 0.5, 1.0, and 1.5 nm with the length kept constant. The perfect kinked nanowire was used as the control. The defect in the 2^nd^ segment has smaller effects on the mechanical properties of KSiNWs than that in the 1^st^ segment. It only affects the relationships between the strain and stress slightly, as shown in Figure S4-S6. And when the width and length of defects were larger than 1.0 nm and 1.5 nm, respectively, the fracture location changed from the middle of KSiNW to kink I.


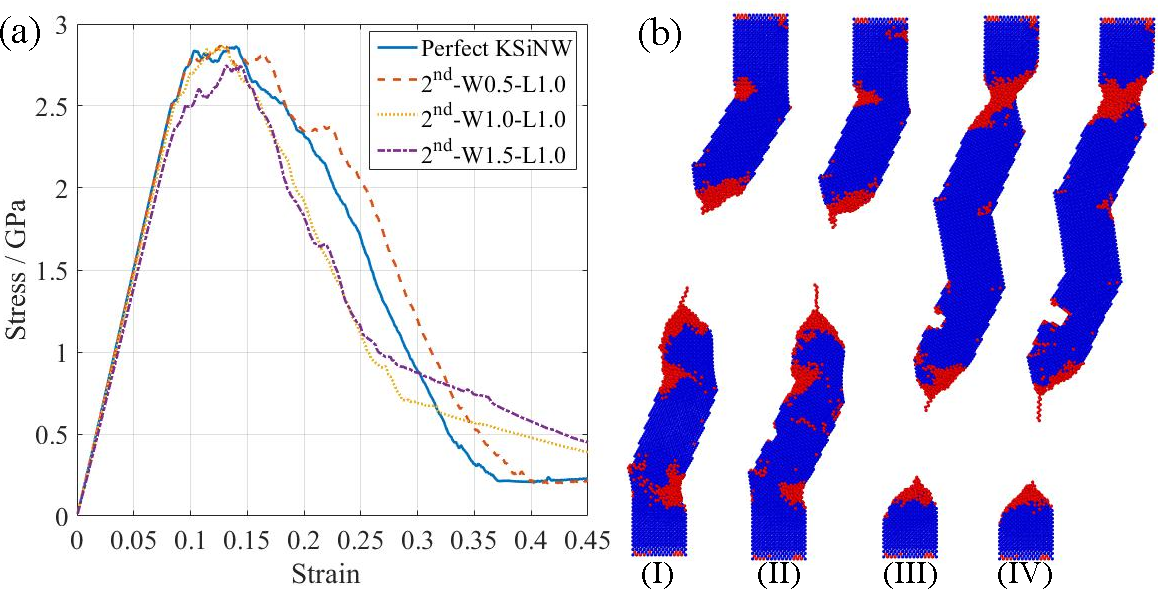


Figure S4. (a) The relationships between the strain and stress of KSiNWs with defects when tensioning. (b) Final profiles after fracture. (I) Control; (II) 2^nd^-W0.5-L1.0; (III) 2^nd^-W1.0-L1.0; (IV) 2^nd^-W1.5-L1.0.


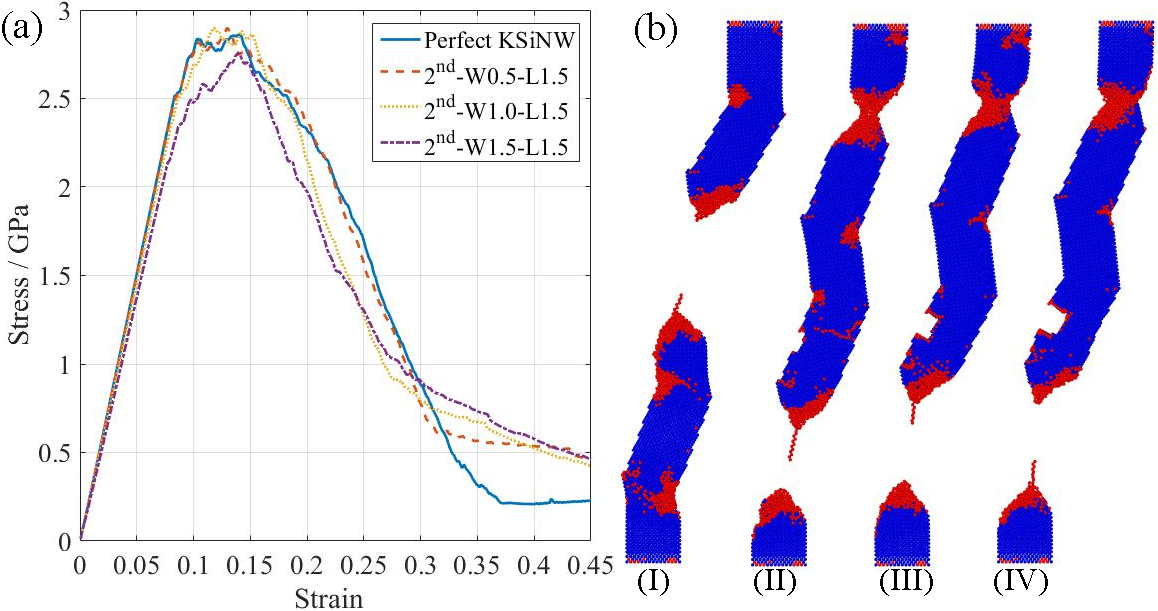


Figure S5. (a) The relationships between the strain and stress of KSiNWs with defects when tensioning. (b) Final profiles after fracture. (I) Control; (II) 2^nd^-W0.5-L1.5; (III) 2^nd^-W1.0-L1.5; (IV) 2^nd^-W1.5-L1.5.


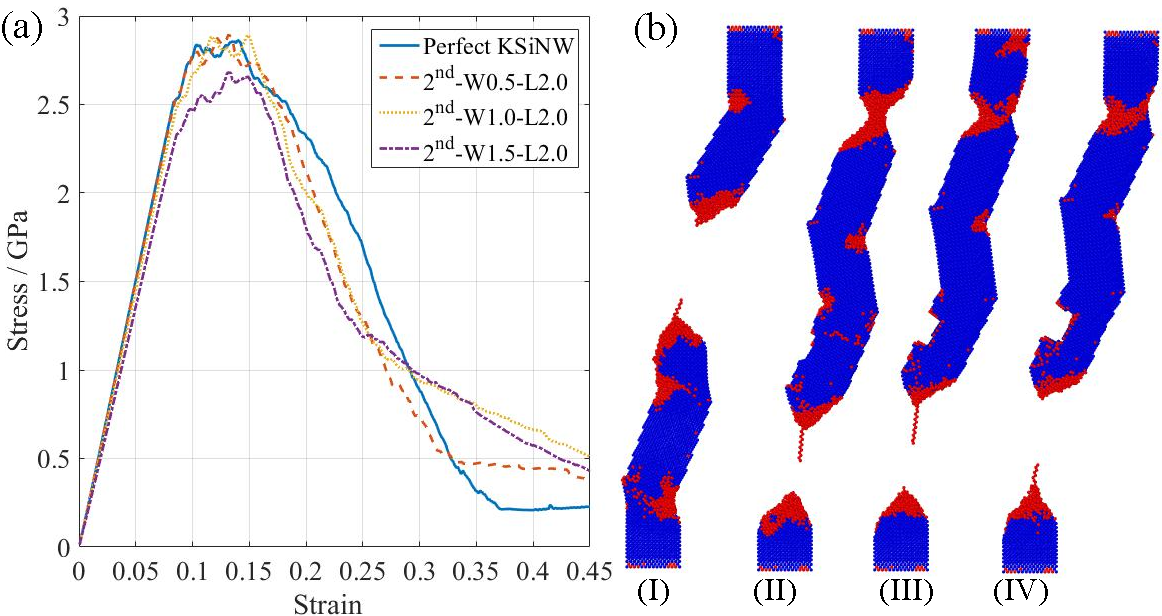


Figure S6. (a) The relationships between the strain and stress of KSiNWs with defects when tensioning. (b) Final profiles after fracture. (I) Control; (II) 2^nd^-W0.5-L2.0; (III) 2^nd^-W1.0-L2.0; (IV) 2^nd^-W1.5-L2.0.

1. The defects located at the 3^rd^ segments. The length (L) of the defect was varied as 1.0, 1.5, and 2 nm with the same width in each case; or the width (W) of the defect was varied as 0.5, 1.0, and 1.5 nm with the length kept constant. The perfect kinked nanowire was used as the control. It can be seen that the defects can notably affect fracture location and the relationship between the strain and stress, especially after yield, as shown in Figure S7-S9. In addition, when the width of defects was larger than 1.0 nm, the yield stress reduced a lot and the fracture location changed from the middle of KSiNW to the point where the defect was.


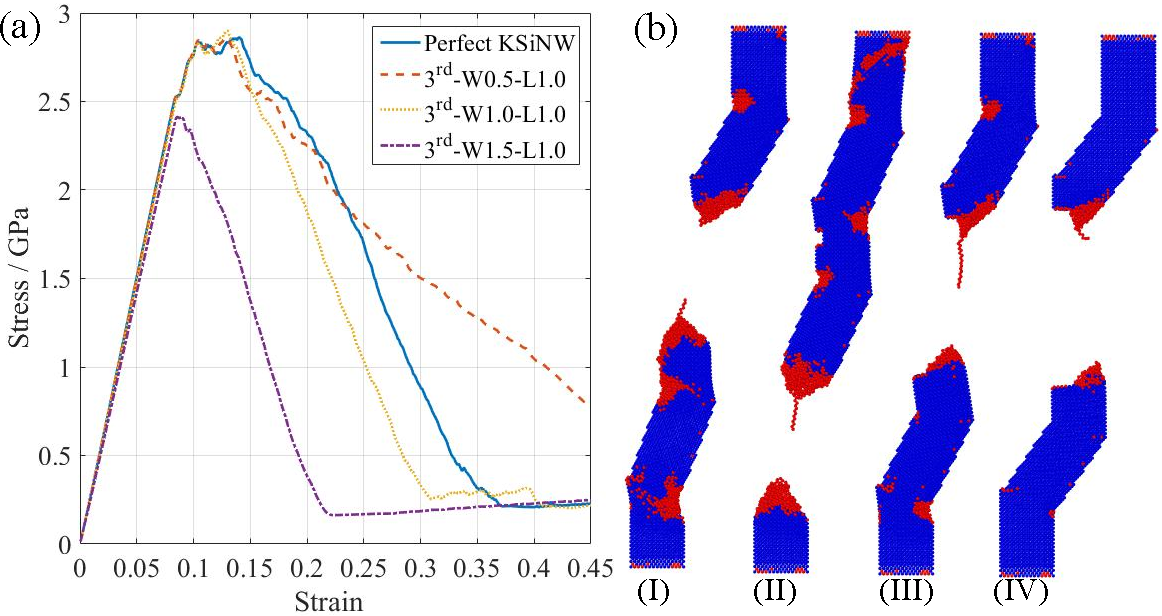


Figure S7. (a) The relationships between the strain and stress of KSiNWs with defects when tensioning. (b) Final profiles after fracture. (I) Control; (II) 3^rd^-W0.5-L1.0; (III) 3^rd^-W1.0-L1.0; (IV) 3^rd^-W1.5-L1.0.


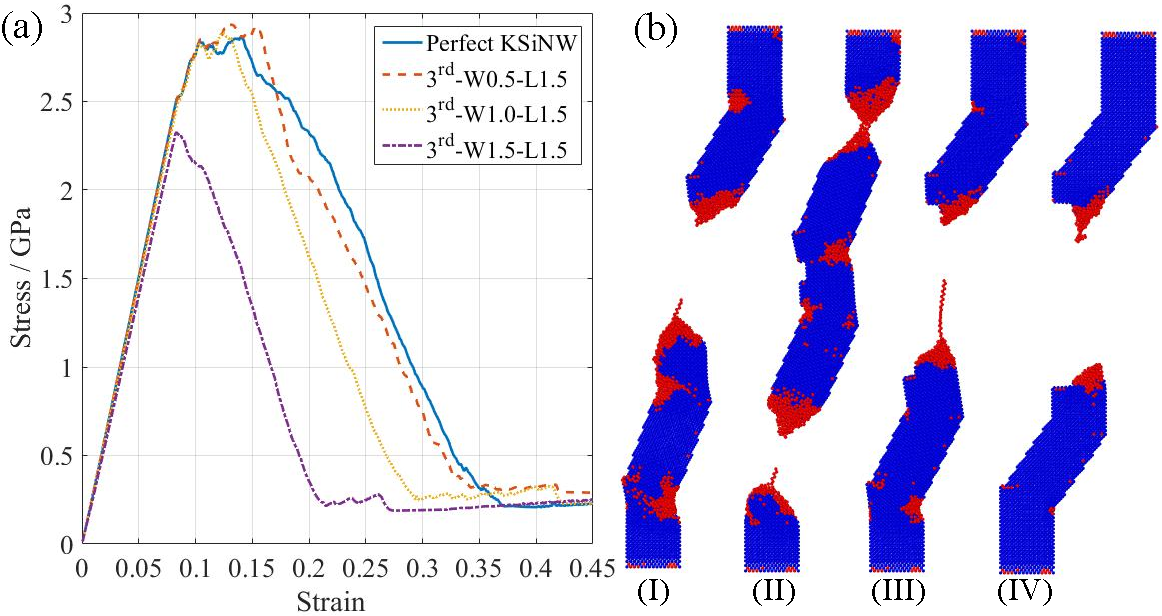


Figure S8. (a) The relationships between the strain and stress of KSiNWs with defects when tensioning. (b) Final profiles after fracture. (I) Control; (II) 3^rd^-W0.5-L1.5; (III) 3^rd^-W1.0-L1.5; (IV) 3^rd^-W1.5-L1.5.


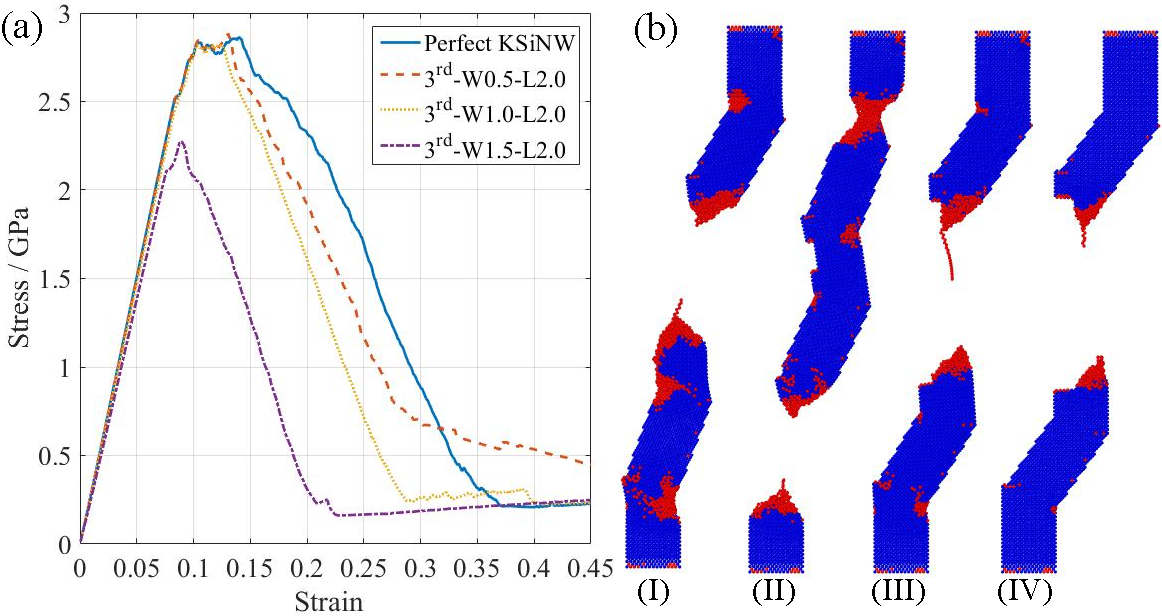


Figure S9. (a) The relationships between the strain and stress of KSiNWs with defects when tensioning. (b) Final profiles after fracture. (I) Control; (II) 3^rd^-W0.5-L2.0; (III) 3^rd^-W1.0-L2.0; (IV) 3^rd^-W1.5-L2.0.

1. The defects located at the 4^th^ segments. The length (L) of the defect was varied as 1.0, 1.5, and 2 nm with the same width in each case; or the width (W) of the defect was varied as 0.5, 1.0, and 1.5 nm with the length kept constant. The perfect kinked nanowire was used as the control. It can be seen that the defects only affect the relationships between the strain and stress slightly and has little effects on the fracture location in the calculated range of defect size, as shown in Figure S7-S9.


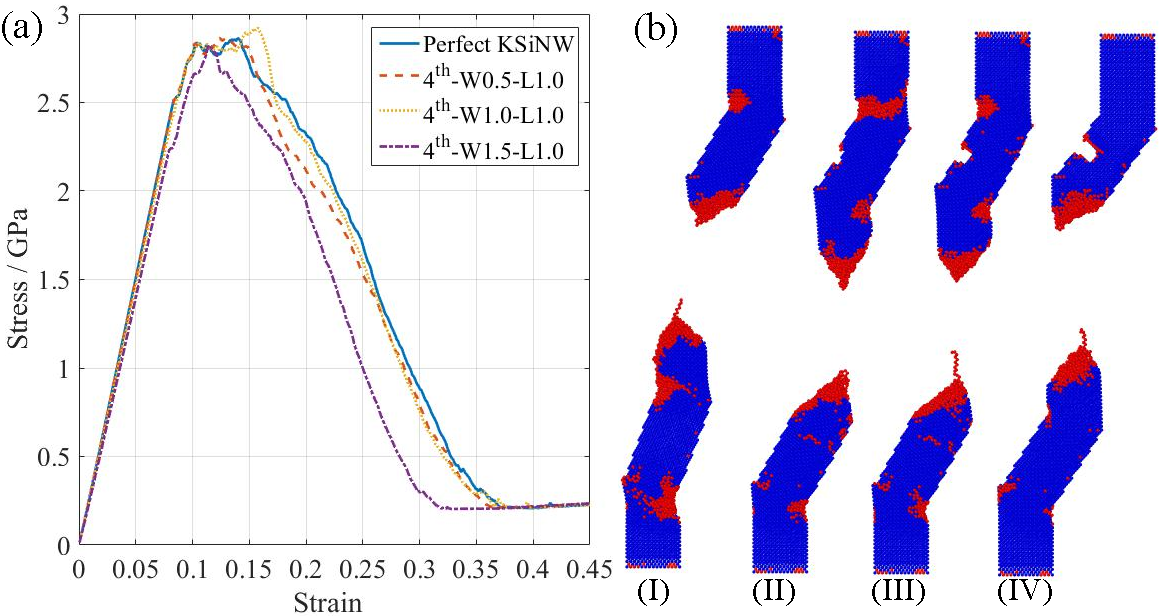


Figure S10. (a) The relationships between the strain and stress of KSiNWs with defects when tensioning. (b) Final profiles after fracture. (I) Control; (II) 4^th^-W0.5-L1.0; (III) 4^th^-W1.0-L1.0; (IV) 4^th^-W1.5-L1.0.


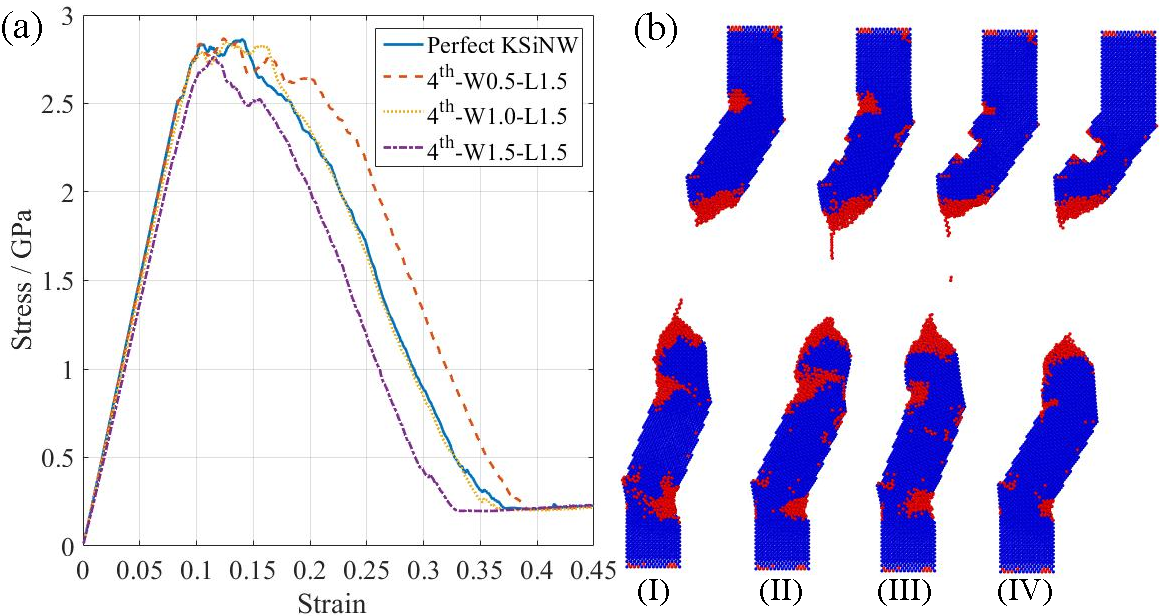


Figure S11. (a) The relationships between the strain and stress of KSiNWs with defects when tensioning. (b) Final profiles after fracture. (I) Control; (II) 4^th^-W0.5-L1.5; (III) 4^th^-W1.0-L1.5; (IV) 4^th^-W1.5-L1.5.


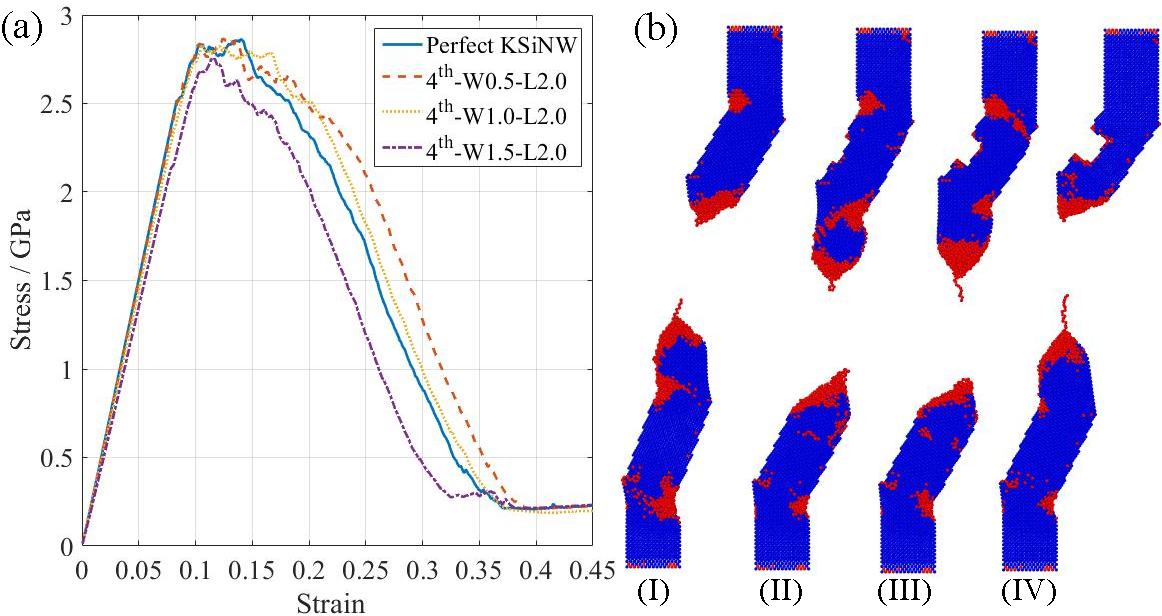


Figure S12. (a) The relationships between the strain and stress of KSiNWs with defects when tensioning. (b) Final profiles after fracture. (I) Control; (II) 4^th^-W0.5-L2.0; (III) 4^th^-W1.0-L2.0; (IV) 4^th^-W1.5-L2.0.
